# Dissociable Effects of Tryptophan Supplementation on Negative Feedback Sensitivity and Reversal Learning

**DOI:** 10.3389/fnbeh.2019.00127

**Published:** 2019-06-28

**Authors:** Martin Thirkettle, Laura-Marie Barker, Thomas Gallagher, Nazgol Nayeb, Luca Aquili

**Affiliations:** Department of Psychology, Sociology and Politics, Sheffield Hallam University, Sheffield, United Kingdom

**Keywords:** tryptophan, serotonin, reversal learning, negative feedback sensitivity, learning

## Abstract

Serotonin has been shown to modulate probabilistic reversal learning (PRL) and negative feedback sensitivity (NFS) in both animal and human studies. Whilst these two measures are tightly coupled, some studies have suggested that these may be mediated by independent mechanisms; the former, representing perseveration and cognitive flexibility, and the latter measuring the ability to maintain a response set (win-stay) at the expense of lose-shift behavior when occasional misleading feedback has been presented. Here, we tested this hypothesis in 44 healthy participants who were administered tryptophan (22 placebo, 22 tryptophan), a precursor to serotonin. We found a dissociable effect of tryptophan supplementation on PRL/NFS. Specifically, tryptophan administration increased NFS compared to the placebo group but had no effect on PRL. We discuss these findings in relation to dosages and with a particular focus on the acute tryptophan depletion (ATD) procedures.

## Introduction

Serotonin (5-HT) has long been implicated in probabilistic reversal learning (PRL) and in processing negative feedback. Both are measured by the PRL task, originally developed in humans (Cools et al., 2002), but then also adapted in animal studies (Bari et al., 2010; Ineichen et al., 2012). In this task, the subject is instructed to choose one of two visual stimuli in order to maximize correct feedback/rewards. Once a certain number of correct responses are made, stimulus-reward contingencies are reversed. Additionally, for a minority of trials (usually 20%), misleading feedback is provided to a normally rewarded response. Negative feedback sensitivity (NFS) is defined as the frequency for which responses shift to the usually non-rewarded choice on such trials. Depressed patients, who are known to have impaired serotonergic function, display normal PRL but higher NFS compared to healthy subjects (Murphy et al., 2003; Taylor Tavares et al., 2008), suggesting that at least behaviorally, these two processes can be decoupled. This effect also appears to be dose and acute/chronic administration dependent; low (acute: 1 mg/kg) doses of the selective serotonin reuptake inhibitor (SSRI) citalopram in rats impaired PRL and increased NFS, whereas high (acute; 10 mg/kg) doses produced the opposite effect. In the same study, chronic high doses (5 mg/kg, daily, 7 days), in contrast, improved PRL but did not affect NFS (Bari et al., 2010; Robbins, 2017). Similar findings for low acute doses of citalopram (30 mg) have also been reported in humans (Chamberlain et al., 2006). Using a different SSRI in rats, escitalopram, all doses (0.03, 0.3 or 1.0 mg/kg) improved PRL but left NFS unaffected (Brown et al., 2012). These findings may partially be explained by the slightly different characteristics of citalopram and escitalopram. Whilst both have similar pharmacokinetics, escitalopram has higher potency and selectivity than citalopram (Carandang et al., 2011). There is also some evidence to suggest that polymorphisms in the gene encoding the serotonin transporter (SERT) can affect NFS and PRL differentially. In this study (den Ouden et al., 2013), individuals who were L’-homozygotes (i.e., decreased levels of extracellular serotonin) had higher NFS than S’ carriers whilst there was no PRL performance difference between these two groups. It is important, nevertheless, to stress that the association between the SERT promoter polymorphism and depression has not been confirmed by a recent meta-analysis (Culverhouse et al., 2018), and may suggest a more complex interaction (Rygula et al., 2018). Studies using the acute tryptophan depletion (ATD) procedure have produced mixed results. Some have reported no effect on PRL (but NFS was not investigated; Evers et al., 2005; Talbot et al., 2006; Finger et al., 2007; van der Plasse and Feenstra, 2008), whereas in others ATD impaired PRL (Murphy et al., 2002). Overall, the studies reviewed on depressed patients, SSRI administration and ATD suggest a potential inverted U-shaped relationship between serotonin concentration and NFS/PRL whereby too low or high 5-HT levels impair performance (Hulsken et al., 2013). Interestingly, a similar relationship has long been reported for the other monoamine neurotransmitter, dopamine (Cools and D’Esposito, 2011).

Here, using a double-blind, placebo-controlled, mixed-design method, we hypothesized that tryptophan supplementation may selectively affect NSF but leave PRL performance intact. Although numerous studies have looked at tryptophan’s modulation of memory, response to unfairness, emotional processing, attention and executive function (Sobczak et al., 2003; Booij et al., 2006; Murphy et al., 2006; Dougherty et al., 2007; Morgan et al., 2007; Silber and Schmitt, 2010; Cerit et al., 2015; Mohajeri et al., 2015), to the best of our knowledge, this is the first study looking at its effects on PRL and NFS.

## Materials and Methods

### Participants

Participants consisted of 44 university students, who were either administered tryptophan (*N* = 22, *M* = 21.4, SD = 3.0, 13 females and nine males) or placebo (*N* = 22, *M* = 20.8, SD = 2.6, 15 females and seven males). The study was approved by the ethics committee of Sheffield Hallam University and complied with the Declaration of Helsinki. Written informed consent was obtained for all participants before testing could take place. Exclusion criteria included: those suffering from cardiac, hepatic, renal and neurological disorders and individuals with a history of alcohol or drug addiction, or psychiatric illness (including individuals who had a history of taking antidepressants). Individuals having a history of taking tryptophan supplements were also excluded.

### Drug Administration

Participants received either 0.8 grams of Tryptophan (supplied by BulkPowders Ltd., Colchester, UK) or 0.8 grams of microcrystalline cellulose (Sigma-Aldrich Co. LLC., St. Louis, MO, USA) dissolved in 200 ml of orange juice as per previously published protocols (Steenbergen et al., 2014). Peak plasma concentrations of tryptophan using this dosage have been shown to occur 60 min following oral administration (Markus et al., 2008).

### Probabilistic Reversal Learning Task

**Figure 1:**
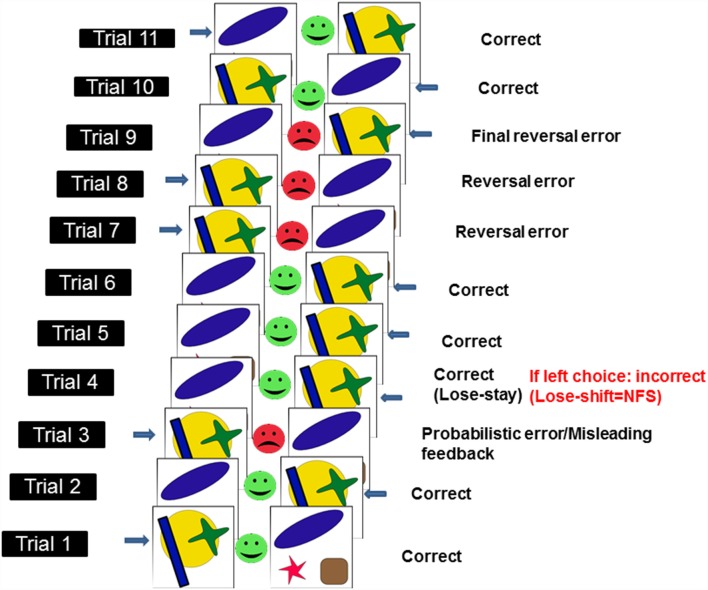
Schematic illustration of probabilistic reversal learning task (PRL) which ran in PEBL. Adapted from Cools et al. (2002). Each pairs of stimuli [i.e., a yellow circle with a green cross and a blue rectangle (here defined as stimulus 1) or a blue oval, a red star and a brown square (here defined as stimulus 2)] represent a trial. Responses (here indicated by a blue arrow next to one of the two stimuli) can either be correct (green smiley feedback face) or incorrect (red frowny feedback face). In a small subset of trials (i.e., <20%), misleading feedback (probabilistic error) is provided to the usually correct and rewarded response (e.g., in this instance, after two correct responses to stimulus 1, the participant is provided with misleading feedback on trial 3). Negative feedback sensitivity (NFS) is measured (in the example above, NFS is measured on the 4th trial) by looking at whether participants stick to the usually rewarded response following negative misleading feedback (lose-stay) or switch (lose-shift). Response reversals (*n* = 10 × 1 block of trials) are required after a series of correct responses.

To assess NFS and reversal learning, we used the PRL paradigm developed by Cools et al. (2002) and which runs in PEBL software (Mueller and Piper, 2014). Here, using trial-and-error feedback, participants need to discover which of two patterns is correct (see Figure 1). To complete the PRL, participants had to finish one block of trials, consisting of 10 reversals. Each block had approximately 150 trials. Each reversal occurred after a variable 10–15 correct responses (including probabilistic errors: here defined as misleading feedback provided to the usually correct and rewarded response). The number of probabilistic errors per reversal varied between 0 and 4. The task was self-paced meaning that there was no timeout period to produce a response in each trial, however, participants were asked to respond as quickly and accurately as possible. Participants were given a full block of practice trials before testing began. We measured the following dependent measures: total errors, reversal errors and NFS. Total errors were made up of incorrect responses occurring before and after each reversal. Reversal errors were counted as the number of incorrect responses after each reversal and before the first correct response following a reversal. NFS was measured by calculating the probability (measured in %) of switching a response following the presentation of misleading negative feedback (i.e., no reward), which occurs in a low proportion of trials. Thus, NFS was high if participants switched response following negative misleading feedback (i.e., lose-shift behavior) and low if participants maintained the usually rewarded option following negative misleading feedback (i.e., lose-stay behavior). See Figure 1 for an illustration of the PRL task.

### Control Measures: Mood and Double-Blinding Efficacy

Transient changes in mood state have been demonstrated to influence cognitive functioning (Federmeier et al., 2001), and of particular relevance to this research, probabilistic learning (Bakic et al., 2014). Therefore, we checked for the potential mood effects induced by tryptophan intake on PRL by administering a computerized adaptation of the visual analog scale (VAS) which was programmed and run in PEBL (Mueller and Piper, 2014), and has previously been used by our research group (Ong Lai Teik et al., 2016; Riby et al., 2017). Seven dimensions of mood/alertness were, recorded with a total minimum score of 7 (low mood/alertness) and a maximum total score of 147 (high mood/alertness). The double-blinding efficacy of placebo/tryptophan administration was checked by a questionnaire given to participants at the end of the experiment.

### Procedure

This was a double-blind, placebo-controlled, mixed design experiment. Participants were required to attend a session lasting approximately 70 min. After screening for eligibility, participants were instructed to refrain from eating/drinking for a minimum of 3 h. They first signed a consent form, followed by the mood questionnaire (VAS; time 1), and were then asked to complete two blocks of the (PRL time 1). They were then randomly assigned to receive either tryptophan or placebo. Sixty minutes following tryptophan intake which corresponds to peak plasma concentration (Markus et al., 2008) or placebo, the VAS and PRL were completed for a second time (VAS time 2; PRL time 2). Following this, participants filled out the tryptophan/placebo double-blind questionnaire, and were debriefed.

### Statistical Analyses

Statistical analyses were performed using SPSS version 23 (SPSS Inc., Chicago, IL, USA). Sample size was chosen to deliver power at 0.8, an alpha level set at 0.05, and a large effect size (*d*) of 0.8 (G*Power 3.1.9.2, Germany). For all the dependent measures of the PRL, we first run an independent sample *t*-test at baseline (time 1), between placebo and tryptophan participants. If there was no significant performance difference at baseline, we calculated a percentage change at time 2 compared with time 1, and used these new values to compare placebo with tryptophan using another independent sample *t*-test. A full breakdown of the raw data is shown in Table 1.

**Table 1:** Mean and SD (in brackets) for negative feedback sensitivity (NFS), reversal errors and total errors in placebo and tryptophan participants.

Group	NFS (%)	Reversal errors	Total errors
Placebo (pre-drug)	48.71 (6.5)	18.20 (6.3)	80.30 (35.6)
Placebo (post-drug)	43.23 (8.2)	17.47 (3.5)	63.68 (31.4)
Tryptophan (pre-drug)	46.34 (4.5)	20.31 (7.0)	76.80 (23.4)
Tryptophan (post-drug)	46.36 (6.1)	17.43 (4.9)	71.00 (35.2)

Mood data were analyzed using a two-way factorial repeated measures analysis of variance (ANOVA), where the factor of Time had two levels (VAS 1–2) and drugs had two (placebo and tryptophan).

The double blinding efficacy of tryptophan/placebo was analyzed using a percentage correct measure. A score of 100 was given if a participant correctly identified condition whereas a score of 0 if not.

## Results

### PRL Negative Feedback Sensitivity (NFS)

To assess the impact of tryptophan administration on NFS, we first took a measurement at baseline (i.e., prior to drug intake). There was no significant difference in NFS performance between placebo and tryptophan participants, *t*_(42)_ = −1.38, *p* = 0.172, *d* = 0.41. Therefore, we calculated a change in performance, expressed in percentage, from time 2 (after drug intake) to time 1 (before drug intake; as in time 2/time 1).

**Figure 2:**
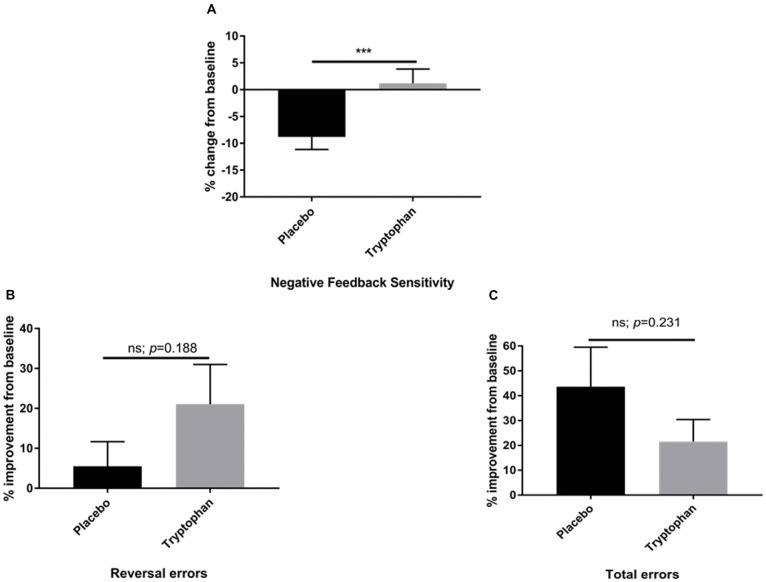
**(A)** Percentage change from baseline (time 2/time 1) for placebo and tryptophan participants with respect to NFS performance. NFS was significantly higher when comparing tryptophan to placebo participants. **(B)** As for **(A)**. There was no significant difference between the two groups on reversal learning errors. **(C)** As for **(A,B)**. There was no significant difference between placebo and tryptophan participants on total errors. ***Indicates *p* < 0.01. Error bars as standard error of the mean (SEM).

Here, whilst there was a reduction in NFS between time 1 and time 2 for placebo participants, tryptophan abolished this change. As a result, NFS was significantly higher for tryptophan than placebo participants, *t*_(42)_ = −2.80, *p* = 0.008, *d* = 0.84, see Figure 2A.

### PRL Reversal Learning Errors

The same analyses were applied for reversal learning errors. At baseline, there was no significant difference in performance between placebo and tryptophan participants, *t*_(42)_ = −1.04, *p* = 0.301, *d* = 0.31.

Both placebo and tryptophan participants improved from baseline. This change in performance from post-drug to pre-drug was not significantly different between the two groups, *t*_(42)_ = −1.33, *p* = 0.188, *d* = 0.40, see Figure 2B.

### PRL Total Errors

As for NFS and reversal learning errors, there was no significant difference between placebo and tryptophan participants at baseline, *t*_(42)_ = 0.34, *p* = 0.703, *d* = 0.11. Tryptophan, also did not affect changes in performance from baseline, *t*_(42)_ = 1.21, *p* = 0.231, *d* = 0.36, see Figure 2C.

### Changes in Reversal Errors Are Independent of Changes in NFS

**Figure 3:**
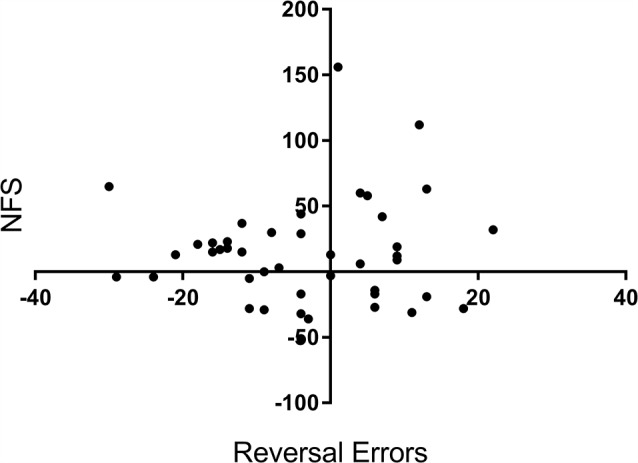
Correlation scatterplot for the variables reversal errors (Y axis) and NFS (X axis) across participants. X and Y values represent a percentage change (%) from baseline (i.e., before drug) to after tryptophan or placebo intake. + for Reversal Errors indicate a reduction in errors, whereas + for NFS equals an increase in NFS.

To confirm that NFS and reversal errors provide separate measures of performance, we run a bivariate correlation analysis between the variables reversal errors (measured as a change in performance from pre-drug to post-drug) and NFS (also measured as a change in performance). There was no significant correlation between reversal errors and NFS, *r* = 0.05, *p* = 0.701, see Figure 3.

### Control Measures: Mood and Double Blinding Efficacy

There was neither a significant main effect of time, *F*_(1,42)_ = 1.04, *p* = 0.313 on mood scores nor a main effect of drugs, *F*_(1,42)_ = 1.45, *p* = 0.234, nor a drugs × time interaction, *F*_(1,42)_ = 0.81, *p* = 0.373. The probability of participants guessing the correct drug (placebo or tryptophan) was at about chance level (56%).

## Discussion

We report a selective effect of tryptophan supplementation on NFS but not on PRL. Whereas for participants in the placebo group their NFS decreased from pre-drug administration to post-drug (due to a practice effect), tryptophan suppressed this reduction. This had the net effect that NFS was higher for participants in the tryptophan condition than the placebo controls (i.e., when measured as a change in performance from baseline to post-drug administration). PRL (reversal errors) and total errors performance, however, was unaffected. Exploratory analyses (i.e., Figure 3), confirm that reversal errors and NFS are dissociated from one another. The observation that reversal and total errors decreased for the tryptophan group from pre-drug to post-drug but NFS did not, demonstrates that tryptophan did not affect the overall acquisition of the task but inhibited NFS learning specifically.

This finding is in line with the tryptophan depletion studies (Evers et al., 2005; Talbot et al., 2006; Finger et al., 2007; van der Plasse and Feenstra, 2008), and suggest that reducing or increasing serotonin, in humans, does not modulate reversal learning. Nevertheless, global serotonin (5-HT) or prefrontal cortex depletion studies in animals and manipulations of serotonin postsynaptically by SSRI (in both animals and humans) demonstrate that serotonin can affect reversal learning (Clarke et al., 2004, 2005; Chamberlain et al., 2006; Bari et al., 2010; Brown et al., 2012), as do studies that target 5-HT receptors (Boulougouris et al., 2008).

Our finding that tryptophan supplementation increased NFS is similar to some reports in which small (but not large) doses of SSRI were administered (Chamberlain et al., 2006; Bari et al., 2010). Interestingly, our data support the idea of a dissociation between PRL and NFS performance as reported in depressed patients and those with serotonergic transporter polymorphisms (Murphy et al., 2003; Taylor Tavares et al., 2008; den Ouden et al., 2013), supporting the view that the PRL is a broader measure of cognitive flexibility than NFS which isolates sensitivity to negative feedback. To this end, ATD studies have highlighted a role for serotonin in aversive processing and punishment prediction (Cools et al., 2008; Robinson et al., 2012). In conclusion, our data provide preliminary evidence for a role of tryptophan supplementation on NFS, an effect that is independent of reversal learning. The current study adds to a complex picture in which general serotonergic activity manipulations, SERT promoter polymorphisms, SSRI administration and targeting of 5-HT receptors, differentially affect NFS and PRL. Future studies that address the SSRI equivalence of the applied tryptophan dosage and its effects on 5-HT levels would be critical, taking into account respective non-linear effects. Although tryptophan supplementation on its own is not going to replace SSRI usage at a therapeutic level, finding out the optimal dosage may support cognitive performance with little to no side effects.

## Data Availability

The datasets for this manuscript are not publicly available because Data can be made available upon request. Requests to access the datasets should be directed to luca.aquili@shu.ac.uk.

## Ethics Statement

The study was approved by the Sheffield Hallam University Psychology Ethics committee. All participants signed a consent form before testing could begin. A set of inclusion/exclusion criteria were used to select participants.

## Author Contributions

L-MB, TG and NN performed the experiments. MT wrote the manuscript. LA designed the experiments, performed the statistical analysis of the data and wrote the manuscript.

## Conflict of Interest Statement

The authors declare that the research was conducted in the absence of any commercial or financial relationships that could be construed as a potential conflict of interest.

## Abbreviations

ATD: acute tryptophan depletion
NFS: negative feedback sensitivity
PRL: probabilistic reversal learning
SERT: serotonin transporter
SSRI: serotonin reuptake inhibitor
VAS: visual analog scale
5-HT: serotonin.
